# Immune Evasion Mechanisms of *Staphylococcus epidermidis* Biofilm Infection

**DOI:** 10.3389/fmicb.2018.00359

**Published:** 2018-02-28

**Authors:** Katherine Y. Le, Matthew D. Park, Michael Otto

**Affiliations:** ^1^Pathogen Molecular Genetics Section, Laboratory of Bacteriology, National Institute of Allergy and Infectious Diseases, National Institutes of Health, Bethesda, MD, United States; ^2^Division of Hospital Internal Medicine, Department of Medicine, Mayo Clinic College of Medicine and Science, Rochester, MN, United States

**Keywords:** *Staphylococcus epidermidis*, biofilm, polysaccharide intercellular adhesin, accumulation associated protein, extracellular matrix binding protein, phenol-soluble modulins, innate immune system, neutrophil

## Abstract

The primary virulence factor of the skin commensal and opportunistic pathogen, *Staphylococcus epidermidis*, is the ability to form biofilms on surfaces of implanted materials. Much of this microorganism’s pathogenic success has been attributed to its ability to evade the innate immune system. The primary defense against *S. epidermidis* biofilm infection consists of complement activation, recruitment and subsequent killing of the pathogen by effector cells. Among pathogen-derived factors, the biofilm exopolysaccharide polysaccharide intercellular adhesion (PIA), as well as the accumulation-associated protein (Aap), and the extracellular matrix binding protein (Embp) have been shown to modulate effector cell-mediated killing of *S. epidermidis*. Phenol-soluble modulins (PSMs) constitute the only class of secreted toxins by *S. epidermidis*, at least one type of which (PSMδ) possesses strong cytolytic properties toward leukocytes. However, through selective production of non-cytolytic subtypes of PSMs, *S. epidermidis* is able to maintain a low inflammatory infection profile and avoid eradication by the host immune system. Taken together, our emerging understanding of the mechanisms behind immune modulation by *S. epidermidis* elucidates the microorganism’s success in the initial colonization of device surfaces as well as the maintenance of a chronic and indolent course of biofilm infection.

## Introduction

Initially described in 1878, staphylococci are Gram-positive microorganisms that have been implicated in infections involving multiple systems of the human body, including the skin and soft tissue, the skeletal system, the respiratory system, the blood stream, and more recently, infections involving implanted medical devices (Lowy, 1998). Staphylococci are further classified as being coagulase-positive, primarily identifying *Staphylococcus aureus* (*S. aureus*), or coagulase-negative (CoNS) (Kloos and Schleifer, 1986). *Staphylococcus epidermidis* (*S. epidermidis*) is the most important and best-studied member of the CoNS group (Vuong and Otto, 2002).

While conventionally regarded merely as an innocuous commensal of the human skin, and increasingly recognized for its beneficial role in skin immunity and within the skin microbiota (Naik et al., 2015; Nguyen et al., 2017), *S. epidermidis* has also emerged as an important human pathogen. This is mostly due to the increased number of implanted prosthetic materials and medical devices (Becker et al., 2014). Lacking most of the many aggressive virulence factors that *S. aureus* produces, *S. epidermidis*’ primary virulence mechanism is to form deeply seated microbial communities, known as biofilms, on implanted medical devices surfaces and native host tissues (Otto, 2008, 2009). For example, *S. epidermidis* is a leading cause of infections on central venous catheters, which occur at a frequency of ∼80,000 annually in the United States alone and may result in severe blood infections (Maki et al., 2006). Furthermore, together with *S. aureus*, it is the premier pathogen causing prosthetic joint infections (Uckay et al., 2009). Finally, *S. epidermidis* causes 15–40% of prosthetic valve endocarditis cases (Wang et al., 2007), a less common but very serious infection. All these infections are easily recognized macro- or microscopically to proceed as biofilm-associated. Within biofilms, *S. epidermidis* are protected from the effects of antimicrobial therapy as well as the host immune system (Vuong and Otto, 2002). Consequently, medical therapy in biofilm-associated infections can be exceptionally challenging (Hoiby et al., 2010), with attempts at infection eradiation often entailing complete removal of the infected foreign body as well as administration of prolonged courses of antimicrobial therapy, approaches that incur risks to patients and excess cost to the health care system (Rogers et al., 2009).

Herein, we attempt to outline our current understanding of the host- and pathogen-derived characteristics that favor effective immune evasion by *S. epidermidis*, such as those contributing toward the microorganism’s successful seeding, colonization of device surfaces, and securing survival in the form of indolent biofilm communities.

## Stages of Biofilm Development

**FIGURE 1:**
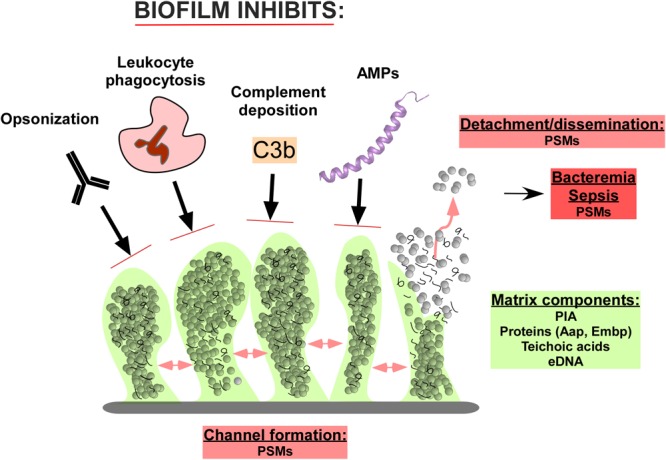
Composition and function of *Staphylococcus epidermidis* biofilms in immune evasion. The biofilm matrix consists of the polysaccharide intercellular adhesion (PIA) exopolysaccharide, proteins such as accumulation-associated protein (Aap) and extracellular matrix binding protein (Embp), teichoic acids, and extracellular DNA (eDNA). Channels in the biofilm are formed by Phenol-soluble modulins (PSMs), which also lead to cell cluster detachment and ultimately dissemination of the infection. Predominantly in immune-compromised individuals, this can lead to bacteremia and sepsis, in which PSMs also likely play a role. The biofilm structure and matrix provides shelter from host defenses, including the binding of opsonizing immunoglobulins, complement components, and antimicrobial peptides (AMPs). Furthermore, the attack of leukocytes is significantly diminished.

Biofilm development has been modeled to occur in three stages: (1) attachment, (2) proliferation/formation of the matured biofilm, and (3) detachment/dispersal (O’Toole et al., 2000; Otto, 2013). During attachment, staphylococcal surface-attached proteins known as microbial surface components recognizing adhesive matrix molecules (MSCRAMMs) establish non-covalent interactions with device surfaces coated by host proteins and host tissues (Patti et al., 1994; Otto, 2008; Joo and Otto, 2015). After attachment, proliferation and maturation of the biofilm follows, with the production of an extracellular matrix consisting of the staphylococcal biofilm exopolysaccharide, polysaccharide intercellular adhesin (PIA) (Mack et al., 1996), also called poly-*N*-acetylglucosamine (PNAG), teichoic acids, proteins, and extracellular DNA (eDNA) (O’Toole et al., 2000; Otto, 2008). During this second stage of biofilm expansion, channels and mushroom-shaped structures form to facilitate nutrient delivery to deeper layers of the biofilm. The last stage of biofilm development is characterized by the detachment and subsequent dispersal/dissemination of biofilm clusters to distal sites (Joo and Otto, 2015), a process mostly due to the activity of the surfactant-like phenol-soluble modulin (PSM) peptides (Wang et al., 2011; Otto, 2013; **Figure 1**).

## PIA/PNAG

**FIGURE 2:**
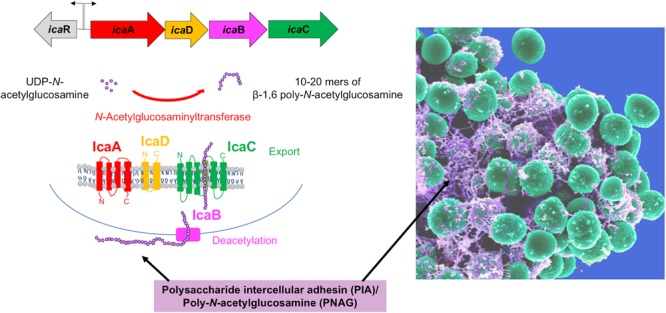
The biofilm exopolysaccharide PIA/PNAG. The PIA biosynthetic locus includes the *icaA* gene, which codes for an *N*-acetylglucosamine (GlcNAc) transferase, which adds GlcNAc residues to a growing poly-GlcNAc chain. IcaD assists in this function in an unknown way. The chain is then believed to be exported by IcaC, because in the absence of *icaC*, polymerization stops at chain lengths of about 10–20 GlcNAc units. IcaB is located at the extracellular cell surface and de-acetylates some of the GlcNAc units, introducing a positive charge in the polymer due to the then unmasked amino groups. This is necessary for PIA surface location (see electron microscopy picture on the right) and functionality in biofilm formation and immune evasion.

The exopolysaccharide PIA/PNAG is an important and abundant component of the *S. epidermidis* biofilm matrix. Initially described in *S. epidermidis* clinical isolates (Mack et al., 1994; Schumacher-Perdreau et al., 1994), PIA is a linear homopolymer of β-1,6-linked *N*-acetylglucosamine monomers, which is positively charged due to partial deacetylation (Mack et al., 1996; Vuong et al., 2004a). These charged moieties facilitate interactions between components of the biofilm extracellular matrix and the staphylococcal cell wall (Mack et al., 1996; Vuong et al., 2004a). PIA is the product of the *icaADBC* operon (Heilmann et al., 1996), which encodes three membrane proteins (IcaA, IcaC, IcaD), and an additional protein (IcaB) that is exported and subsequently attached to the staphylococcal cell surface by non-covalent interactions (Vuong et al., 2004a). IcaA, an *N-*acetylglucosaminyltransferase, along with IcaD, catalyzes the conversion of UDP-*N*-acetylglucosamine to 10–20-mers of β-1,6-linked poly-*N*-acetylglucosamine (Gerke et al., 1998). IcaC is needed to further extend the *N-*acetylglucosamine oligomers, and probably represents a PIA export system (Gerke et al., 1998), while IcaB deacetylates the poly-*N-*acetylglucosamine molecule (Vuong et al., 2004a), enabling localization of the PIA molecule to the cell surface (**Figure 2**).

## Accumulation-Associated Protein (Aap) and Extracellular Matrix Binding Protein (Embp)

PIA does not appear to be absolutely required for *S. epidermidis* biofilm formation, as *S. epidermidis* isolates from biofilm-associated catheter and prosthetic joint infections were found to be negative for the *ica* genes (Francois et al., 2003; Chokr et al., 2007; Rohde et al., 2007). In *S. epidermidis ica(-)* strains, intercellular adhesion has been shown to be mediated through proteinaceous components, of which two of the best-defined are the accumulation-associated protein (Aap) (Hussain et al., 1997) and the extracellular matrix binding protein (Embp) (Christner et al., 2010). However, PIA-dependent biofilms appear to be more structured and robust than those dependent on proteins (Schommer et al., 2011).

Accumulation-associated protein is a 240-kDa surface-bound protein consisting of two domains, designated as A and B. Proteolytic cleavage of domain A induces a conformation change in the protein, enabling domain B to mediate polymerization of Aap into fibrils, leading to aggregation and biofilm formation in the PIA-negative *S. epidermidis* clinical strain 5179 (Rohde et al., 2005; Conrady et al., 2008; Conlon et al., 2014).

Extracellular matrix binding protein is a 1-MDa protein surface protein that has been implicated as an intercellular adhesin that mediates *S. epidermidis* biofilm formation. Embp is composed of 59 found-in-various-architecture (FIVAR) domains and 38 G-related albumin-binding (GA) domains (Christner et al., 2010). *In vitro*, the FIVAR domains were observed to bind to fibronectin, thus suggesting a role in the initial attachment phase of biofilm development. Furthermore, like the FIVAR domains described in the *S. aureus* protein Fmtb, which interacts with *N-*acetylglucosamine, the FIVAR domains of Embp in *S. epidermidis* are hypothesized to also bind to PIA (Christner et al., 2010). However, it appears that FIVAR domains alone are insufficient for biofilm aggregation (Christner et al., 2010).

## Phenol Soluble Modulins (PSMs)

Phenol soluble modulins (PSMs) were first described in 1999 in *S. epidermidis* (Mehlin et al., 1999). Later it was found that *S. epidermidis* produces six PSM peptides, PSMα, PSMβ1, PSMβ2, PSMδ, PSM𝜀, and δ-toxin (Otto et al., 2004; Yao et al., 2005). Next to δ-toxin, β-PSMs are the primary PSMs produced in *S. epidermidis* (Yao et al., 2005; Cheung et al., 2010). Besides these PSMs, which are all core genome-encoded, there is one PSM, PSM-mec, which is encoded within some types of the methicillin resistance-conferring mobile genetic element, SCC*mec* (Queck et al., 2009). *S. epidermidis* β-PSMs (Wang et al., 2011) have been shown to be key effector molecules in biofilm structuring and dissemination (Otto, 2013), but investigation in *S. aureus* indicates all PSMs have similar capacities (Periasamy et al., 2012). The general mechanism by which PSMs contribute to biofilm structuring and dispersal is believed to be the disruption of non-covalent (hydrophobic, electrostatic) interactions between biofilm matrix macromolecules (Otto, 2013). The PSM structuring effect occurs independently of the mode of biofilm formation (PIA-dependent or -independent) (Wang et al., 2011).

## Components of the Innate Immune Response in Staphylococcal Infections

In immunocompetent hosts, the primary innate immune response against planktonic staphylococcal infections involve the complement system as well as effector cells (Foster, 2005). The complement system’s primary role is to recruit effector molecules that label staphylococci and mark them for destruction by effector cells. In addition, the activated complement system produces a cell-killing membrane attack complex. Complement fixation occurs through the classical, alternative, and lectin pathways. While the alternative and lectin pathways are part of the innate immune system, activation of the classical pathway requires binding of an antibody to an antigen on the staphylococcal surface. Activation of these pathways leads to the generation of C3a, a pro-inflammatory chemoattractant, which recruits phagocytes to the infection site. While the alternative pathway seems to play a smaller role in host defense against *S. epidermidis* infections (Fredheim et al., 2011), the classical and lectin pathways are believed to be necessary for rapid killing of planktonic *S. epidermidis* by effector cells (Kristian et al., 2008).

Neutrophils (or, polymorphonuclear leukocytes, PMNs) have been identified as the primary effector cells in the innate immune defense against staphylococcal infection. Activation of neutrophils might involve: (1) recognition of pathogen-associated molecular patterns (PAMPs) on bacterial surfaces by the cellular recognition receptors (Toll-like receptors), (2) deposition of opsonins on the bacterial surface, or (3) the direct action of complement system (Rigby and DeLeo, 2012). Leukocyte responses involve direct contact of the staphylococci with the host immune cells, and are channeled via pattern recognition receptor-dependent pathways (Flannagan et al., 2015). While the role of these pathways in staphylococcal infection have primarily been established in *S. aureus*, there is evidence that the cellular recognition receptor, Toll-like receptor 2 (TLR2), plays a significant role in *S. epidermidis* bloodstream infection (Strunk et al., 2010). However, how exactly pathogen recognition receptors impact *S. epidermidis* blood infection remains unknown.

Once activated, neutrophil-mediated killing involves reactive oxygen species as well as non-oxygen-dependent processes involving antimicrobial peptides (AMPs), such as defensins and cathelicidins, and antimicrobial proteins, such as lysozyme (Hancock and Diamond, 2000; Nauseef, 2007). Again, much of this is general knowledge obtained in other bacteria, but a significant role of AMP resistance mechanisms, such as proteolysis by the SepA protease (Cheung et al., 2010) and PIA-mediated resistance on the cell surface (Vuong et al., 2004b) (see below), as well as sensing of the presence of AMPs by the *S. epidermidis* ApsRSX system (Li et al., 2007), have been demonstrated to contribute to *S. epidermidis* survival in neutrophils and biofilm infection.

## The Innate Immune Response to *S. epidermidis* Biofilm Infection

As compared to *S. aureus*, infections by *S. epidermidis* are characterized by proceeding with decidedly less inflammation and overall morbidity. This is also true for biofilm-associated infections. Specific mechanisms that *S. epidermidis* employs to limit the inflammatory response will be discussed in the following. On the other hand, there are specific interactions between *S. epidermidis* and the host that can be described as pro-inflammatory, which will also be discussed. These may be less important given the overall outcome.

Early research has suggested that biofilm formation protects *S. epidermidis* from phagocytosis by effector cells (Johnson et al., 1986; Heinzelmann et al., 1997). When compared to the response against planktonic infection, the innate immune response against *S. epidermidis* biofilm infection has been characterized as being less pronounced (Cerca et al., 2006; Kristian et al., 2008; Schommer et al., 2011; Thurlow et al., 2011; Hanke et al., 2012, 2013; Spiliopoulou et al., 2012). However, vigorous induction of the complement system has been described (Kristian et al., 2008). Yet, deposition of C3b and IgG on *S. epidermidis* biofilm surfaces was paradoxically diminished, when compared to the planktonic mode of growth, suggesting that subsequent activation and killing of *S. epidermidis* biofilm by effector cells might also be impaired (Kristian et al., 2008).

Diminished activation of leukocytes has been described in *S. epidermidis* biofilm infection and seems to involve multiple mechanisms. First, observation of a blunted response to pro-inflammatory compounds by macrophages after exposure to *S. epidermidis* biofilm suggests that interference of signaling by cellular recognition receptors might partially contribute to the observed quiescent immune response toward biofilms, when compared to the planktonic mode of growth (Schommer et al., 2011). Additionally, when rabbit polyclonal PIA/PNAG antiserum was utilized as opsonin, fewer deaths of *S. epidermidis* derived from disrupted biofilms were detected, when compared to their isogenic planktonic form (Cerca et al., 2006), implicating opsonin deposition as one aspect of innate immune response that may be modulated in *S. epidermidis* biofilm infection.

Moreover, it has been hypothesized that sufficient contact of the bacteria with components of the host immune system is necessary for efficient activation of effector cells (Schommer et al., 2011). In fact, deficient uptake of bacteria by macrophages and reduced generation of an NF-κB-mediated macrophage inflammatory response have been described in *S. epidermidis* biofilm (Riber et al., 1995; Schommer et al., 2011), phenotypes probably linked to the immune evasion effect of PIA (see below) and biofilm formation *per se*. Additionally, selective modulation of the immune response seems to occur, as discriminatory activation of the weakly pro-inflammatory J774A.1 macrophage by *S. epidermidis* biofilms was reported (Schommer et al., 2011). Once consumed by macrophages, biofilm-derived *S. epidermidis* appears to be able to survive more effectively within these effector cells, than their isogenic planktonic counterpart (Spiliopoulou et al., 2012).

## The Role of PIA/PNAG

The exopolysaccharide PIA represents a particularly important constituent of the microorganism’s immune evasion strategies (Foster, 2005; Otto, 2009). When compared to the isogenic 1457 *ica(-)* M10 strain, *S. epidermidis* 1457 is killed by PMNs less efficiently (Vuong et al., 2004b; Kristian et al., 2008), likely by reducing phagocytosis. Reduced uptake has been shown directly for macrophages (Schommer et al., 2011). This is likely mostly due to PIA being a preeminent biofilm constituent and a molecule that leads to the formation of bacterial aggregates. However, there are also more specific interactions of PIA with the immune system that have been reported. Some of them are probably due to PIA forming a sort of positively charged “capsule” around *S. epidermidis*, which is a general mechanism to shield the bacteria from immune recognition. In accordance with that notion, differences in PMN-mediated killing have been attributed to decreased availability of opsonizable surfaces in *ica(+)* biofilm (Cerca et al., 2006) and differences in PIA-mediated opsonization mechanisms (Kristian et al., 2008). Furthermore, PIA appears to prevent neutrophil attacks when cell clusters are disintegrated (Vuong et al., 2004b).

Animal experiments as well as observations from human *S. epidermidis* biofilm infections have largely recapitulated an overall attenuated immune response to biofilm infection due to wildtype *S. epidermidis* strains when compared to those derived from the isogenic *ica(-)* isolates (Fey et al., 1999; Rupp et al., 1999a,b; Vuong et al., 2004a). Levels of inflammatory cytokines (including TNF-α, IL-6, IFN-γ) and CD11b expression in human whole blood were higher in infections with *S. epidermidis* 1457 *ica(-)* M10 than those with the isogenic wildtype strain (Fredheim et al., 2011). Moreover, skin and soft tissue inflammatory changes surrounding subcutaneous catheter insertion sites were found to be more severe in mice infected with wildtype *S. epidermidis* compared to the *ica(-)* strain (Kristian et al., 2008), which correlates with *in vitro* findings of differential activation of the complement pathways (Kristian et al., 2008). Furthermore, levels of IL-6 in blood cultures of *ica(+) S. epidermidis* isolated from neonates were lower as compared to that of an *ica(-)* control strain (Hartel et al., 2008). Finally, there was a significant correlation between low levels of C-reactive protein (CRP) and *in vitro* biofilm-forming capacity in *S. epidermidis* isolates from neonate blood infection (Klingenberg et al., 2005).

Moreover, PIA confers protection against the action of host AMPs (Vuong et al., 2004b). The mechanism behind the protective effect of PIA against AMPs is thought to involve repulsion between the cationic PIA and the commonly cationic AMPs. However, a similar effect was reported in the same study for the anionic AMP dermcidin, suggesting that different mechanisms of PIA-mediated resistance to AMPs also exist (Vuong et al., 2004a; Otto, 2006).

Polysaccharide intercellular adhesion has also been reported to act in a pro-inflammatory fashion. For example, it has been implicated in the activation of the complement system during infection, based on the comparison of wild-type 1457 and isogenic *ica(-)* M10 strains (Fredheim et al., 2011). This phenotype was also achieved using a PIA preparation in the same study. These results await verification by a demonstration of a specific receptor-mediated mechanism.

## The Role of Aap and Embp

In contrast to PIA, which forms a meshwork of extracellular matrix that embeds adjacent *S. epidermidis* cells, Aap is covalently attached to the *S. epidermidis* surface through its LPXTG motif and extends radially to form tuffs of fibrils (Rohde et al., 2005; Banner et al., 2007). Embp is also found on the *S. epidermidis* surface, forming a proteinaceous matrix (Schommer et al., 2011). Like in a predominantly PIA-dependent biofilm, biofilms dependent on Aap or Embp protect *S. epidermidis* from J774A.1 macrophage phagocytosis (Christner et al., 2010; Schommer et al., 2011).

## The Role of PSMs

Complete genome analyses of *S. epidermidis* have shown that besides from their crucial roles in *S. epidermidis* biofilm structuring and dissemination, PSMs are the only gene products with cytolytic properties in *S. epidermidis* (Zhang et al., 2003; Gill et al., 2005), with the PSMδ of the α-type PSM subclass discovered as the first highly potent cytolysin produced by *S. epidermidis* (Cheung et al., 2010, 2014). PSMδ has been shown to be highly cytolytic against human neutrophils (Cheung et al., 2010). Nevertheless, when culture filtrates of *S. epidermidis* were examined, low levels of neutrophil lysis were measured (Cheung et al., 2010), and this is thought to be a consequence of selective production of PSMs by *S. epidermidis*, favoring production of the non-cytolytic β-type PSMs over the α-type PSMs, with the exception of the δ-toxin (PSMγ) (Cheung et al., 2010). This strategy results in a low inflammatory profile and likely contributes toward *S. epidermidis’* successful immune evasion and subsequent colonization of device surfaces (Cheung et al., 2010).

The most frequent and serious complication of biofilm-associated infection on indwelling medical devices is bloodstream infection that arises from hematogenous seeding of dispersed biofilm clusters, which can develop into full-blown sepsis. As the most frequent cause of biofilm infections on such devices, *S. epidermidis* is also a leading cause of hospital-associated bloodstream infections, in particular in neonates (Cheung and Otto, 2010; Becker et al., 2014). Believed for the longest time to be due to an over-reacting immune response to invariant bacterial surface structures such as lipoteichoic acids and lipopeptides (Bochud and Calandra, 2003), a recent study has implicated PSM-mec, which is highly expressed in many methicillin-resistant isolates of *S. epidermidis* (Qin et al., 2017). Like other PSMs, PSM-mec is pro-inflammatory due to activation of the formyl peptide receptor 2 (Kretschmer et al., 2010). Most likely, other PSMs have similar effects, which remain to be determined.

Finally, it needs to be mentioned that there are reports on the presence of other toxins in *S. epidermidis*, such as a pathogenicity-island encoded enterotoxin, but this has to be considered a very rare exception (Madhusoodanan et al., 2011). Reports claiming much more widespread presence of toxins in *S. epidermidis* have to be regarded as due to inappropriate species identification.

## Conclusion and Outlook

In summary, by modulating multiple aspects of the host innate immune response using secreted exopolysaccharides, peptides, and other proteinaceous biofilm components, *S. epidermidis* is able to orchestrate an overall low inflammatory profile, escape killing by the host innate immune system, and persist on the surfaces of implanted devices (**Figure 1**). While often considered innocuous partly due to its low inflammatory infection profile, it is precisely the low immunogenicity that makes *S. epidermidis* difficult to diagnose at initial stages of infection. Often, *S. epidermidis* biofilm infections are diagnosed at the third stage of biofilm infection, when infection sequelae are more severe and difficult to manage and treat, suggesting a need for a more complete basic understanding of the mechanisms behind this relatively quiescent immune response, so that safer avenues of therapy as well as novel approaches to prevention might be pursued.

Presently, our knowledge on the pro-inflammatory capacity of *S. epidermidis* in comparison to *S. aureus* during infection is poor. Future research should include an in-depth analysis of how only recently discovered pro-inflammatory molecules of *S. epidermidis*, such as the PSMs, contribute to inflammation and immune priming. This research will also benefit from a comparison with recent advances in the understanding of how *S. epidermidis* primes the skin immune system during the commensal state (Naik et al., 2015; Linehan et al., 2018).

## Author Contributions

All authors listed have made a substantial, direct and intellectual contribution to the work, and approved it for publication.

## Conflict of Interest Statement

The authors declare that the research was conducted in the absence of any commercial or financial relationships that could be construed as a potential conflict of interest.
